# Magnitude and risk factors of non-communicable diseases among people living with HIV in Tanzania: a cross sectional study from Mbeya and Dar es Salaam regions

**DOI:** 10.1186/1471-2458-14-904

**Published:** 2014-09-02

**Authors:** Gibson B Kagaruki, Mary T Mayige, Ester S Ngadaya, Godfather D Kimaro, Akili K Kalinga, Andrew M Kilale, Amos M Kahwa, Godlisten S Materu, Sayoki G Mfinanga

**Affiliations:** National Institute for Medical Research-Tukuyu Center, P.O. Box 538, Tukuyu Mbeya, Tanzania; National Institute for Medical Research-Muhimbili Center, Dar es Salaam, Tanzania; Centre for International Health, University of Bergen, Bergen, Norway

**Keywords:** Non-communicable diseases, Risk factors, ART, People living with HIV, Tanzania

## Abstract

**Background:**

HIV and Non communicable diseases (NCDs) are major problem of public health importance in developing countries. This study was conducted to explore and establish information on the magnitude, distribution of NCDs risk factors among people living with HIV (PLWHIV) which is scarce in Tanzania.

**Method:**

A cross sectional study was conducted to PLWHIV from 12 care and treatment clinics in Dar es Salaam and Mbeya regions from October 2011 to February 2012. Data on demographic characteristics, NCD risk factors including behavioral, biochemical tests and physical measurements was collected from PLWHIV.

**Results:**

Of 754 PLWHIV recruited, 671(89.0%) consented to participate in the study and 354/671(52.8%) were on antiretroviral therapy (ART). The following NCD risk factors: raised blood levels of low density lipoprotein (61.3% vs 38.7%, p < 0.001) total cholesterol (TC) (71.6% vs 28.4%, p < 0.001) and triglyceride (67.0% vs 33.0%, p = 0.001) as well as overweight/obesity (61.1% vs 38.9%, p = 0.010), abnormal waist circumference (61.7% vs 38.3%, p < 0.001) and being aged >40 years (63.3% vs 36.7%, p < 0.001) were more prevalent among PLWHIV on ART than ART naïve. The prevalence of Diabetes mellitus among PLWHIV was 4.2% and was slightly high among those ART naïve (4.7% vs 3.7%). The prevalence of hypertension was 26.2% and was high among those on ART (30.0% vs 21.9%, p = 0.010). Being aged >40 years (AOR = 2.52, 95% CI 1.37-4.63), abnormal waist circumference (AOR = 2.37 95% CI 1.13-5.00), overweight/obesity (AOR = 2.71, 95% CI 1.26-5.84) and male sex (AOR = 1.17, 1.02-4.20) were the predictors of hypertension among patients on ART while raised TC (AOR = 1.47 (1.01-2.21) and being aged >40 years (AOR = 3.42, 95% CI 2.06-5.70) were predictors for hypertension among ART naïve patients.

**Conclusion:**

This study has revealed that the magnitude of NCD risk factors is significantly higher among PLWHIV on ART than those not on ART. Initiating and strengthening of interventions for minimizing preventable NCD risks should be considered when initiating ART among PLWHIV. Regular monitoring of NCD risk factors is of paramount importance among ART patients.

## Background

According to the recent survey, the prevalence of HIV among sexual active aged 15–49 years in Tanzania; is 5.1% [1]. The prevalence has consistently continued to be high in urban than rural settings [1–3]. However, the overall HIV prevalence among individuals aged 15–49 years has declined from 7% (2003–04) to 6% (2007–08) to 5.1% (2011–12) [1–3]. The prevalence varied greatly across regions, ranging from 2.0% to 16.0% (2007–08) and from 1.5% to 14.8% (2011–12) [1, 3]. The declining of the overall HIV prevalence has been associated with behavioral change, availability and improved accessibility of ART services and biomedical interventions programs targeting both the general population and specific vulnerable groups [4].

Studies have reported an increased risk of cardiovascular disease (CVDs) among people living with HIV (PLWHIV). This is due to the linkage between HIV/AIDS and non-communicable diseases (NCDs) [5–7]. The linkage is either due to direct effects of HIV or indirectly by anti-retroviral therapy (ART) regimens. HIV can directly damage the cells of blood vessels and contribute to atherosclerosis, or hardening of the arteries [8]. This is due to inflammation induced by HIV infection or its associated proteins which may promote atherosclerosis and formation of high-risk plaque, thus increasing the risk of myocardial infarction and stroke [9–11]. Several studies have found that the various drugs included in ART regimens have different effects on patients’ cholesterol and fatty acid balance [9, 11]. The therapy seems to affect the risks of heart diseases by increasing cholesterol levels and changing fat distribution, particularly increasing abdominal fat [12–14]. For example nucleoside reverse transcriptase inhibitors (NRTIs), stavudine mainly causes increased blood levels of triglycerides (TG), low density lipoprotein cholesterol (LDL-C) and total cholesterol (TC) [15, 16]. All of the protease inhibitors (PIs) except atazanavir have been associated with hyperlipidemia, for example ritonavir-boosted PIs, cause elevations of LDL, TC and TGs and a decrease in high density lipoprotein cholesterol (HDL-C) [17, 18]. The prevalence of hypertriglyceridemia and hypercholesterolemia among PLWHIV on PI-based antiretroviral regimen has been documented to 38.2% and 25% respectively and it appeared to be dose- and probably time-related [19]. Overweight and obesity as other risk factors for CVDs has been reported to be common morphological alteration among PLWHIV on ART [20]. Being overweight or obese is well known traditional risk factor associated with raises blood pressure (BP) and dyslipidemia [21].

A study conducted in Nigeria revealed an increase of prevalence of hypertension from 26% to 31.7% after two years of being initiated on ART [22]. In Botswana, the prevalence of raised BP was twice as much among PLWHIV compared to the general population [23]. The incidence of diabetes mellitus is fourfold among HIV-infected men on ART compared to HIV negative men [24]. HIV infection has also been associated with incidence of stroke, for example, the HIV prevalence among patients with stroke in Malawi was 48% [25] and in Tanzania was 20.9% and the mean age was lower among stoke patients with HIV infection compared to their HIV negative counterparts [26].

Kidney disease is an important contributor to HIV/AIDS related morbidity and mortality. As the life expectancy of HIV-infected patients increases with the use of ART, chronic medical conditions such as renal failure are increasingly prevalent [27]. In addition, the risk of type2 diabetes mellitus is increased among PLWHIV and is associated with cumulative exposure to ART [24, 28]. Furthermore, in many areas dietary counseling for HIV is tailored towards increased calorie intake and weight gain, hence, majority consume fatty diets to increase their weight; coupled with ART induced dyslipidemia, these patients are at an increased risk of an uncontrolled weight gain which is a risk factor for NCDs [29]. Due to the evidences reported elsewhere much information related to NCDs to PLWHIV in Tanzania is needed to inform various HIV programs. The scientific evidence to substantiate the foregoing discussion in linking occurrence of NCDs in HIV patients on ART is limited in Tanzania. The situation on the magnitude and NCD risk factors among PLWHIV in Tanzania is not known hence posing challenges for designing, planning and implementing possible interventions to the affected population. Therefore, this study was proposed to obtain findings to inform policy on the magnitude and risk factors of NCDs among PLWHIV for strengthening interventions to prevent morbidity and mortality in patients which might be accelerated by co-morbidities (NCD and HIV).

## Methods

### Study area, design and population

A cross-sectional study was conducted in two purposefully selected regions namely Mbeya and Dar es Salaam. The former representing rural settings and the latter is the largest urbanized city in Tanzania. The two regions had the largest number of PLWHIV enrolled in care and treatment clinics (CTCs) in the country [30]. PLWHIV aged 18 years and above attending 12 CTCs in these regions were interviewed using a structured questionnaire. Anthropometric measurements and biochemical profile were also assessed. The study population was both PLWHIV on ART and those not on ART.

### Sample size calculation

To calculate sample size, we used prevalence (P) of Diabetes Mellitus among PLWHIV admitted with stroke at Muhimbili National Hospital i.e. 11.1%; hence the prevalence of patients with no diabetes (as our 1-P) was 88.9% [26]. Using randomly sampling formula, with the following parameters: Z = the level of statistical significant set up at the level of 95% confidence interval (1.96) and (e) = likelihood error (2.5%) then the minimum sample size obtained was 602. To take care of non-responders, 20% non response was considered during sample size calculation; leading to a minimum sample size of PLWHIV attending CTC services to be include in the study to be 754.

### Sampling procedures

Two districts were randomly selected from each region: Ilala and Kinondoni districts from Dar es Salaam and Kyela and Rungwe districts from Mbeya region. A list of the CTCs arranged by strata/level (dispensary, health center and hospital) was made available per site and from each stratum one CTC was randomly selected using a randomly selection Table [31]. A total of 12 CTCs (three from each district) were therefore selected. Then, separate lists of PLWHIV on ART and those not on ART were made for each of these CTCs. The lists provided a sampling frame for both PLWHIV on ART and those not on ARTs. The proportion allocation statistical technique which is described by randomly selection table [31] was used to obtain sub-sample from different levels of health facilities i.e. dispensaries, health centers and hospitals from each district. The names of CTCs from each district with respective sub actual sample size in the brackets are as follows: - Kinondoni district were Mwananyamala (91), Sinza (92) and Mwenge (3); Ilala district were Amana (95), Buguruni (44) and Mnazimmoja (28); Rungwe district Tukuyu(149), Masukulu(5) and Mwakaleli (14) and from Kyela district were Kyela(155), Njisi(9) and Ngonga(6).

### Ethical issue

Protocol for this study was reviewed and approved by the Medical Research and Coordinating Committee(MRCC) of the National Institute for Medical Research (NIMR)-Tanzania (certificate No NIMR/HQ/R.8a/Vol.IX/1130). The study involved PLWHIV attending CTCs which are the clinics dedicated for offering services to PLWHIV only. The study team (which was formed mainly by medical personnel) worked closely with the CTC administration. Each study subject, was individually and privately informed on the aim and purpose of the study, risks and benefits of participating in the study, and that, his/her participation was purely voluntary, and there would be no any negative effect that she/he will get for not consenting to participate in the study. All questions and queries were satisfactorily answered and clarified before consenting through signing a written informed consent form. Confidentiality was maintained throughout the study period. Apart from the research team and the CTC staff, data was not accessed by any other person. Each study participant was informed of his/her physical assessment and biochemical test results and given appropriate health education. In addition, every participant was given a hard copy of the feedback form for both physical measurements and biochemical tests performed. If the study participant was found to have either NCD or NCD risk factor, the information was communicated to the CTC in-charge for further evaluation, assessment, management and monitoring according to the WHO guidelines [32].

### Data collection

A structured questionnaire with both closed and open-ended questions was adopted from STEPs survey tool. Adopted tool was translated from English to Swahili language which is the national and familiar language to the intended subjects. The questionnaire was back-translated to English to see if the intended message was maintained. Before commencing the actual data collection a three days training was conducted to the data collectors and majority were medical personnel; in day one for familiarizing the study aims and protocol, day two for piloting the tool and day three for refining the tool. The refined tool was used for data collection. Information on demographic characteristics, NCD risk factors, anthropometric and biochemical measurements such as blood glucose, lipid profile was collected from each study subject. At every CTC, an equal number of study subjects on ART and those not on ART were obtained at exit point i.e. dispensing table. All physical measurement data (weight, height, waist circumference and blood pressure) was collected by the research team members while the samples for biochemical test (TC, HDL, LDL, TG and FBG) were collected and analyzed by qualified laboratory technicians.

### Physical measurements

#### Blood pressure (BP) measurements

Blood pressure was measured while patients were in a sitting position, using an M4 Omron® automatic blood pressure device. The blood pressure reading was consistently taken from the left arm, three times at 3 minutes interval [33]. The average of the two last readings was estimated and used in the analysis. High blood pressure was classified as BP of ≥140/90 mmHg.

#### Weight measurement

Weight measurement was taken using a SECA® weighing scale on a flat hard surface. Patients were instructed to remove any heavy clothing (such as coats) and shoes and stand still on the weighing scale, with hands by their sides. The weighing scales were calibrated daily according to manufacturer’s instructions.

#### Height measurement

Height was measured with a SECA® stadiometer, while a patient is facing directly ahead. Patients were instructed to remove their shoes, caps or head scarfs, keep their feet together, and stand with their arms by the sides. Measurement was taken with heels, buttocks and upper back in contact with the stadiometer.

#### Body mass index (BMI)

The BMI was calculated as weight (in Kilograms) divided by height squared (m^2^). The BMI results were categorized as: obesity if the BMI was ≥30 kg/m^2^; Overweight if the BMI was > 25 but < 30 kg/m^2^, and normal if the BMI was between 18-25 kg/m^2^ and underweight if below 19 kg/m^2^ [34].

#### Waist circumference

The measurement was made at the approximate midpoint between the lower margin of the last palpable rib and the top of the iliac crest as per WHO guidelines [35]. We used the European cut off to interpret the waist circumference measurements as per World Health Organizations (WHO) and International Diabetes Federation (IDF) recommendations. According to the above guidelines the range of abnormal waist circumference of male and female are >94 cm and >80 cm respectively [36, 37].

### Laboratory tests

In addition to the questionnaires and anthropometric measurements, the study subjects were requested to return to the clinics to provide fasting blood samples for blood glucose and cholesterol measurements in the following morning. Patients were provided with return transport fare Tanzanian Shillings 3,000/= (equivalent to 2 US$) to facilitate the return visits. All patients were provided with a feedback of their results and advised accordingly.

### Fasting blood glucose measurement

Patients were instructed to fast for at least 8 hours before the test. All tests were performed by trained laboratory technicians. Blood sample was taken by finger prick (capillary) and measured using Hemoque® 201 analyzer. Diagnosis of diabetes mellitus was done according to WHO classification [38].

### Lipid profile estimations

Blood was taken for lipid measurements namely including: High Density Lipoprotein Cholesterol (HDL-C), Low Density Lipoprotein Cholesterol (HDL-C), Total Cholesterol (TC), and triglycerides Cholesterol (TG-C). Blood sample of 4mls for lipid profile were taken from a venipuncture and placed in a plain vacutainer. Blood was kept at room temperature and was analyzed the same day, within hours of sample collection. Samples were analyzed using fully automated biochemistry analyzers by the direct end point enzymatic method.

### Analysis

Data collected was double entered and coded using Epidata Version 3.1. Data was then exported to SPSS version 18 for Windows (SPSS Inc, Chicago, IL, USA) and STATA version 12 (STATA Corp Inc., TX, USA) for cleaning and analysis. Pearson Chi square statistics test was used to compare group differences for categorical variables. Associations and relationships were considered statistically significant if p < 0.05. Logistic regression was used for modeling multiple risks for hypertension and it was also intended for the diabetes mellitus; however, analysis for diabetes mellitus was not conducted because of the collected small number of diabetes cases. Crude and Adjusted Odds Ratios (OR) with 95% confidence intervals (CI) were reported. Variables that were considered significant in then univariate analysis were analyzed using multivariate analysis. Backward stepwise selection with removal testing was used, which based on probability of the likelihood ratio statistic. The significance level of a likelihood ratio statistic was compared to a cut-off value of 0.1. Twelve variables were considered in the initial model in each group. For the patients on ART only four out of six variables which were significant in the univariate analysis were considered significant in the multivariate analysis based on the p-value for likelihood ratio (-2 Log likelihood = 45.97, p = 0.001) with common odds ratio of 3.34(2.00-5.58). While for the non ART patients only two out of four variables which were significant in the univariate analysis were significant in multivariate analysis based on the p-value for likelihood ratio (-2 Log likelihood = 24.19, p < 0.001) with common odds ratio of 2.37(1.31-4.31).

## Results

### General characteristics

The demographic characteristics of the study participants are shown in [Table 1]. A total of 754 PLWHIV were recruited for the study, however, 671(89.0%) participants consented and participated in the study. Patients on ART and female constituted 52.8% (354/671) and 70.5% (473/671) of the study participants respectively. The overall mean age of the respondents in years (SD) was 38.7 (10.1). The mean age of males was 42.5(11.1) and female was 37.1(9.2). The mean age for patients on ART was 40.6(9.3) and 36.7(10.6) for those not on ART. Age groups 18–34 and 35–44, constituted a majority of study population, each forming 37.4%. Three quarters of the respondents had attained primary level of education and more than half (53.0%) were married.

**Table 1:** **Social and demographic characteristics of PLWHIV by ART status (n = 671)**

Characteristics	Both, n (%)	Patients on ART, n (%)	Patients not on ART, n (%)
**Response rate**	671/754(89.0)	354/377(93.9)	317/377(84.1)
**Region**			
Dar es Salaam	336(50.1)	179(50.6)	157(49.5)
Mbeya	335(49.9)	175(49.4)	160(50.5)
**Age group**			
18-34	251(37.4)	100(28.2)	151(47.6)
35-44	251(37.4)	147(41.5)	104(32.8)
45-54	117(17.4)	75(21.2)	42(13.2)
55+	52(7.7)	32(9.0)	20(6.3)
**Sex**			
Male	198(29.5)	114(32.2)	84(26.5)
Female	473(70.5)	240(67.8)	233(73.5)
**Education level**			
No formal education	84(12.5)	44(12.4)	40(12.6)
Adult education	3(0.4)	2(0.6)	1(0.3)
Primary education	503(75.0)	265(74.9)	238(75.1)
Secondary education	69(10.3)	37(10.5)	32(10.1)
College/University	12(1.8)	6(1.7)	6(1.9)
**Marital status**			
Single	39(12.3)	83(12.4)	44(12.4)
Married	168(53.0)	319(47.5)	151(42.7)
Separated	30(9.5)	74(11.0)	44(12.4)
Divorced	10(3.2)	17(2.5)	7(2.0)
Widow/Widower	53(16.7)	136(20.3)	83(23.4)
Cohabiting	17(5.4)	42(6.3)	25(7.1)
**Occupation**			
Farmer	234(34.9)	115(35.5)	119(39.5)
Self-employed	224(33.4)	134(41.4)	90(29.9)
Employed	76(11.3)	36(11.1)	40(13.3)
Others	91(13.6)	39(12.0)	52(17.3)

### Magnitude of non communicable diseases (NCD) risk factors

Prevailing risk factors for NCD among the participants included: low blood levels of HDL (71.9%), consumption of fruits or vegetables below the recommended standards (70.0%), poor participation on vigorous physical activities (including running, cycling, digging, manual construction works, brisk walking) (47.8%), abnormal waist circumference(46.6%) and raised blood levels of LDL (42.9%) [Table 2].

**Table 2:** **Association between prevalence of NCD risk factors and ART status**

Risk	Both n (%)	Patients on ART, n (%)	Patients not on ART, n (%)	χ ^2^	P-value**
LDL (>2.6 mmol/l)	282/658(42.9)	173(61.3)	109(38.7)	13.68	p < 0.001
HDL (<1.03 mmol/l for Male, < 1.29 for Female)	473/658(71.9)	206(43.6)	267(56.4)	61.49	p < 0.001
TC (>6.2 mmo/l)	215/658(32.7)	154(71.6)	61(28.4)	44.3	p < 0.001
Triglycerides (≥2 mmol/l)	109/658(16.6)	73(67.0)	36(33.0)	10.18	0.001
Overweight/Obesity (BMI ≥ 25 kg/m^2^)	175/671(26.2)	107(61.1)	68(38.9)	6.68	0.01
Waist circumference (male ≥94 cm/female >80 cm)	303/650(46.6)	116(38.3)	187(61.7)	15.83	p < 0.001
Aged >40 years	275/671(41.0)	174(63.3)	101(36.7)	20.67	p < 0.001
^1^Smoking	26/671(3.9)	10(38.5)	16(61.5)	2.22	0.99
^2^Alcohol	221/671(32.9)	106(48.0)	115(52.0)	3.04	0.048
Consumed vegetables/fruits <5 days in a week	470/671(70.0)	240(51.1)	230(48.9)	1.81	0.104
^3^Never participate on vigorous intensity activity	321/671(47.8)	168(52.3)	153(47.7)	0.04	0.448

**Comparison between patients on ART and those not on ART.

^1^Smoking: Current tobacco users (smoke and smokeless tobacco users).

^2^Alcohol: Used alcohol in the last 12 months.

^3^Never participate on vigorous intensity activity: included not participating on running, cycling, digging, manual construction works, brisk working).

### Association between the magnitudes of NCD risks factors and ART use

Low blood levels of HDL and current alcohol drinking habit was significantly more observed among participants not on ART compared to those on ART (56.4% vs 43.6%, p < 0.001) and (52.0% vs 48.0%, p = 0.048) respectively. The proportion of patients on ART with raised blood levels of LDL was high compared to those not on ART (61.3% vs 38.7%, p < 0.001). Prevalence of raised blood levels of TC was almost three times among participants on ART compared to those not on medications (71.6% vs 28.4%, p < 0.001). The proportion of patients on ART with raised Triglyceride and who were overweight/obese was significantly high compared to those not on ART (67.0% vs 33.0%, p = 0.001) and (61.1% vs 38.9%, p = 0.010) respectively. Patients on ART were older compared to the counterpart group (those aged >40 yrs, 63.3% vs those aged ≤40 yrs 36.7%, p < 0.001) and patients on ART with abnormal waist circumference were almost two times than the counterpart group (61.7% vs 38.3%, p < 0.001) [Table 2].

### Magnitude of NCD co-morbidities among PLWHIV

#### Hypertension

The prevalence of hypertension (blood pressure of ≥140/90 mmHg measured during the study period or previously diagnosed with hypertension) among study participants was 26.2% (175/668). The prevalence of hypertension was significantly higher among those on ART than those not on ART (30.0% vs 21.9%, p = 0.011). Further analysis i.e. univariate and multivariate logistic regression analysis was conducted to establish the relationship between the prevalence of raised BP and the risk factors of the disease [Table 3].

**Table 3:** **Multivariate logistic regression analysis to assess relationship between prevalence of hypertension and NCD risk factors among HIV positive patients utilizing CTC services**

Risk factor	Patients not on ART			Patients on ART		
	COR	AOR	P-value*	COR	AOR	P-value*
^1^Smoking	2.40(0.68-8.46)			1.20(0.37-3.85)		
^2^Alcohol	1.26(0.78-2.03)			1.27(0.74-2.17)		
Consumed vegetables/fruits <5 days in a week	0.79(0.49-1.28)			0.84(0.47-1.51)		
^3^Never participate on vigorous intensity activity	1.43(0.90-2.25)			0.71(0.41-1.22)		
LDL (>2.6 mmol/l)	1.46(0.92-2.32)			1.42(0.81-2.47)		
Aged >40 years	3.20(1.97-5.18)	3.42(2.06-5.70)	p < 0.001	2.59(1.50-4.49)	2.52(1.37-4.63)	0.003
HDL (<1.03 mmol/l Male, < 1.29 Female)	1.06(0.66-1.69)			1.79(0.71-4.44)		
Triglycerides (≥2 mmol/l)	1.96(1.22-3.16)			0.94(0.49-1.83)		
Waist circumference (male ≥94 cm/female >80 cm)	1.64(1.03-2.62)			2.86(1.64-5.00)	2.37(1.13-5.00)	0.023
Overweight/Obesity (BMI ≥ 25 kg/m^2^)	1.95(1.21-3.16)			3.85(2.14-6.93)	2.71(1.26-5.84)	0.011
Sex (Male)	0.87(0.53-1.42)			1.52(0.85-2.70)	1.17(1.02-4.20)	0.043
TC (>6.2 mmo/l)	2.01(1.26-3.20)	2.28(1.39-3.75)	0.001	0.86(0.43-1.72)		
-2LR		24.19	p < 0.001		45.97	0.001
Common odds ratio		3.34(2.00-5.58)			2.37(1.31-4.31)	

*P-Value for AOR, ^1^Smoking: Current tobacco users (smoke and smokeless tobacco users).

^1^Smoking: Current tobacco users (smoke and smokeless tobacco users).

^2^Alcohol: Used alcohol in the last 12 months.

^3^Never participate on vigorous intensity activity: included not participating on running, cycling, digging, manual construction works, brisk working).

#### Diabetes mellitus (DM)

The prevalence of Diabetes mellitus (DM) i.e. fasting blood glucose (FBG) of ≥6.1 mmol/l (measured during the study period or previously diagnosed with DM among PLWHIV was 4.2% (28/671). The prevalence of DM among participants on ART and not on ART was 3.7% and 4.7% respectively.

### Multivariate analysis for multiple risk factors for hypertension by ART use

Univariate and Multivariate analysis on the risk factors for hypertension by ART status among PLWHIV is shown on [Table 3]. With multiple regression analysis, age above 40 years (AOR = 3.42, 95% CI 2.06-5.70) and raised blood TC (AOR = 1.47 (1.01-2.21) were the risk factors that predicted the prevalence of hypertension among participants not on ART. On the other hand, age above 40 years (AOR = 2.52, 95% CI 1.37-4.63), abnormal waist circumference (AOR = 2.37 95% CI 1.13-5.00), overweight/obesity (AOR = 2.71, 95% CI 1.26-5.84), and a male sex (AOR = 1.17, 1.02-4.20) predicted the prevalence among those on ART.

## Discussion

The most common NCD risk factors observed among both groups (on ART and not on ART) participants of this study were: low blood levels of HDL, consumption of fruits or vegetables below the recommended standard days per week, poor participation on vigorous physical activities, abnormal waist circumference and raised blood levels of LDL. Further, findings from this study showed that NCDs risk factors were more prevalent among PLWHIV on ART than those not ART. This could partly be due to the effects of the ART drugs itself, as some of the ART drugs, for example, stavudine has been linked with raised blood levels of TG, LDL-C and raised TC [15, 16]. Moreover, all PIs except atazanavir have been associated with hyperlipidemia. For example ritonavir-boosted PIs, has been associated with elevated blood levels of LDL, TC and TGs and a decrease in HDL-C [17, 18]. Effect of ART on the blood lipids levels was also observed in study conducted in Uganda, in which, low blood levels of HDL was observed in 60% of patients on ART, raised blood levels of TC on 39% and raised levels of TG and LDL on 24% and 20% respectively [39]. In South Africa the prevalence of obese/overweight among PLWHIV was observed to be 33% and 58% before and after initiation of ART respectively [40].

On the other hand, advanced age by itself is also associated with NCDs. This could have also contributed on the higher prevalence of NCD risk factors among study participants on ART compared to those not on ART. This is because this analysis showed that participants aged above 40 years were more among those on ART than their counterparts. Various studies conducted elsewhere on PLWHIV have reported aging as a traditional and remerging NCD risk factor and especially after initiation of ART since PLWHIV are surviving longer up to the NCD risk age level [41–43].

Findings from this analysis showed that the prevalence of hypertension was significantly higher among participants on ART (30%) as compared to those not on ART (21.9%). Similar findings were observed in Nigeria where there was an increase of the prevalence of hypertension from 26% to 31.7% after two years of ART initiation [22]. The high prevalence of the disease among participants on ART may be due to the observed excessive risk factors like raised blood levels of LDL, TC, triglyceride, overweight/obesity and advanced age (i.e. >40 years) among this group than their counterparts. Similar observations have been reported in other studies, in which advanced age, overweight and obesity, and abnormal waist circumference, among PLWHIV who are on ART, have been associated with hypertension [20, 44]. Furthermore, the ARTs are associated with heart diseases by increasing blood levels of cholesterol and changing fat distribution, particularly increasing abdominal fat [12–14]. However, the prevalence of hypertension of 26.2% observed in our study among patients on ART is lower compared with 45% that was observed in Malaysia [44]. In this analysis, the prevalence of DM was slightly high among participants not on ART compared to those on ART (4.7% vs 3.7%). This observation is different from the observations reported from other studies, in which the prevalence of DM was reported to be more among patients on ART compared to those not on ART [39, 45, 46]. However, participants on ART in our study may need to be followed up and assessed on their status of DM in the future, as such risk factors as, prevalence of hypertension, raised blood levels of LDL, TC, and triglycerides as well as overweight, obesity and abnormal waist circumference, all of which are also the risk factors for DM, were observed more among participants on ART than those not on ART. Further, with efforts to expand HIV programmes services, PLWHIV are initiated on ART, consequently they are living longer and ageing, and are developing non-HIV-related chronic conditions similar to the rest of the population [42, 43]. Lastly, in this study no further analysis i.e. Univariate and Multivariate analysis were conducted to establish relationship between diabetes mellitus and associated risk factors because the number of observed cases of diabetes was small.

## Conclusion

This study revealed that the prevalence of hypertension and diabetes mellitus among PLWHIV was high. The prevalence of hypertension was significantly higher among participants on ART than those not on ART. Majority of the NCD risk factors including raised blood levels of LDL, TC and triglyceride also other risk factors were abnormal waist circumference, overweight/obesity and advanced age (i.e. >40 years) were more prevalent among patients on ART than those not on ART. Based on these findings, we recommend the initiation and strengthening of interventions on health education and awareness creation on NCDs and NCD risk factors at each CTC. We also recommend regular monitoring and screening for NCD and risk factors to PLWHIV attending CTC for early detection of NCDs and NCDs risk factors to prevent HIV-NCDs co-morbidities and mortality. Continuous monitoring and screening of PLWHIV at CTC will also provide evidence to programme managers and policy makers to inform planning and decision making.

### Limitations of the study

Our study design was cross sectional, which is strong to establish associations but weak in generating causal relationship between variables [47]. We therefore did not include causal relationships as this requires longitudinal cohort study design. In this study, univariate and multivariate analysis to establish relationship between diabetes and associated risk factors was not conducted because the number of observed cases was small. This may have affected the quality and the impact of the intended message of our study. Furthermore, during data analysis we encountered the problem of missing data especially the biochemical data. This was associated with poor turn-up in the next day of the interview for the biochemical test even though the participants were assured of return fare. In addition, the study ended up with moderate response rate of 89% and is presumed to be contributed by patients who were not on ART as they tended to refrain from taking part into the study in claim that they did not have time. They considered that they did not require extra services as compared to their counterparts who were on ART. Lastly, the study lacked equal gender representation as more than two quarters (70.5%) of the study respondents were female. However, challenges including gender norms around masculinity and sexuality, confidentiality problems, self stigma and fearing of community stigma and discriminations were the most documented bottlenecks which hinder men from utilizing care and treatment services in many countries including Tanzania [48, 49].

## References

[1] Tanzania Commission for AIDS (TACAIDS), Zanzibar AIDS Commission (ZAC), National Bureau of Statistics (NBS), Office of the Chief Government Statistician (OCGS), and ICF International (2013). Tanzania HIV/AIDS and Malaria Indicator Survey 2011–12.

[2] Tanzania Commission for AIDS (TACAIDS), National Bureau of Statistics (NBS), and ORC Macro (2005). Tanzania HIV/AIDS Indicator Survey 2003–04.

[3] Tanzania Commission for AIDS (TACAIDS), Zanzibar AIDS Commission (ZAC), National Bureau of Statistics (NBS), Office of the Chief Government Statistician (OCGS), and Macro International Inc (2008). Tanzania HIV/AIDS and Malaria Indicator Survey 2007–08.

[4] URT (2009). National Multisectoral HIV Prevention Strategy 2009–2012 ‘Towards achieving Tanzania without HIV.

[5] Rabkin M, Nishtar S (2011). Scaling up chronic care systems: Leveraging HIV programs to support noncommunicable diseases services. J Acquir Immune Defic Syndr.

[6] Hirschhorn LR, Kaaya SF, Garrity PS, Chopyak E, Fawzi MCS (2012). Cancer and the 'other' noncommunicable chronic diseases in older people living with HIV/AIDS in resource‒limited settings: A challenge to success. AIDS.

[7] Nigatu T (2012). Integration of HIV and Noncommunicable Diseases in Health Care Delivery in Low- and Middle-Income Countries. Prev Chronic Dis.

[8] Eliseo A, Morgello ES, Klotman ME, Mosoian A, Lento PA, Berman JW, Schecter AD (2008). Human Immunodeficiency Virus (HIV) Infects Human Arterial Smooth Muscle Cells in Vivo and in Vitro: Implications for the Pathogenesis of HIV-Mediated Vascular Disease. Am J Pathol.

[9] Hsue PY, Giri K, Erickson S, MacGregor JS, Younes N, Shergill A, Waters DD (2004). Clinical features of acute coronary syndromes in patients with human immunodeficiency virus infection. Circulation.

[10] de Saint Martin L, Vandhuick O, Guillo P, Bellein V, Bressollette L, Roudaut N, Amaral A, Pasquier E (2006). Premature atherosclerosis in HIV positive patients and cumulated time of exposure to antiretroviral therapy (SHIVA study). Atherosclerosis.

[11] Segev A, Cantor WJ, Strauss BH (2006). Outcome of percutaneous coronary intervention in HIV-infected patients. Catheter Cardiovasc Interv.

[12] Andrew C, Samaras K, Burton S, Law M, Freund J, Chisholm DJ, Cooper DA (1998). A syndrome of peripheral lipodystrophy, hyperlipidaemia and insulin resistance in patients receiving HIV protease inhibitors. AIDS.

[13] Kotler DP (2008). HIV and antiretroviral therapy: lipid abnormalities and associated cardiovascular risk in HIV-infected patients. J Acquir Immune Defic Syndr.

[14] Currier J, Scherzer R, Bacchetti P, Heymsfield S, Lee D, Sidney S, Tien PC (2008). Regional adipose tissue and lipid and lipoprotein levels in HIV-infected women. J Acquir Immune Defic Syndr.

[15] Riddler SA, Li X, Chu H, Kingsley LA, Dobs A, Evans R, Palella F, Visscher B, Chmiel JS, Sharrett AR (2007). Changes in serum lipids among HIV-infected men on highly active antiretroviral therapy. HIV Med.

[16] Crane HM, Grunfeld C, Willig JH, Mugavero MJ, Van Rompaey S, Moore R, Rodriguez B, Feldman BJ, Lederman MM, Saag MS, Kitahata MM (2011). Impact of NRTIs on lipid levels among a large HIV-infected cohort initiating antiretroviral therapy in clinical care. AIDS.

[17] Michael DP, Stein JH, Aberg JA, Fichtenbaum CJ, Gerber JG, Tashima KT, Henry WK, Currier JS, Sprecher D, Glesby MJ (2003). Guidelines for the Evaluation and Management of Dyslipidemia in Human Immunodeficiency Virus (HIV)–Infected Adults Receiving Antiretroviral Therapy: Recommendations of the HIV Medicine Association of the Infectious Disease Society of America and the Adult AIDS Clinical Trials Group. Clin Infect Dis.

[18] Dau BT, Holodniy M (2008). The Relationship between HIV Infection and Cardiovascular Disease: Current Cardiology Reviews. Academic Journal.

[19] Savès M, Raffi F, Capeau J, Rozenbaum W, Ragnaud JM, Perronne C, Basdevant A, Leport C, Chêne G (2002). Factors related to lipodystrophy and metabolic alterations in patients with human immunodeficiency virus infection receiving highly active antiretroviral therapy. J Acquir Immune Defic Syndr.

[20] Hejazi N, Lee MHS, Geok Lin K, Lee C, Choong K (2010). Factors Associated with Abdominal Obesity among HIV-infected Adults on Antiretroviral Therapy in Malaysia. Global Journal of Health Science.

[21] Crum CN, Tejidor R, Medina S, Barahona I, Ganesan A (2008). Obesity among Patients with HIV. The Latest Epidemic AIDS Patient Care and STDs.

[22] Denue BA, Muazu PJ, Gashau W, MBO DN, Ajayi NA (2012). Effects of highly active antiretroviral therapy (HAART) on blood pressure changes and its associated factors in HAART naive HIV-infected patients in north eastern Nigeria. Archives of Applied Science Research.

[23] Dusara P, Bussmann H, Lima C, Tsalaile L, Makhema J, Campa A, Widenfelt E, Sales S, Li Y, Burns PJ, Marlink R, Baum MK (2009). **Predictors of hypertension among HIV infected adults in Botswana, Africa** [J]. Faseb J.

[24] Brown TT, Cole SR, Li X, Kingsley LA, Palella FJ, Riddler SA, Visscher BR, Margolick JB, Dobs AS (2005). Antiretroviral therapy and the prevalence and incidence of diabetes mellitus in the multicenter AIDS cohort study. Arch Intern Med.

[25] Kumwenda J, Mateyu G, Kampondeni S, Van Dam P, Lvan L, Zijlstra E (2005). Differential diagnosis od stroke in a setting of high HIV prevalence in Blantyre Malawi. Malawi Med J.

[26] Mlayi M, Bakari M (2010). The prevalence of HIV among patients admitted with stroke at the Muhimbili National Hospital, Dar es Salaam, Tanzania. Tanzan J Health Res.

[27] Schwartz EJ, Szczech LA, Ross MJ, Klotman ME, Winston JA, Klotman PE (2005). Highly active antiretroviral therapy & the epidemic of HIV + end-stage renal disease. J Am Soc Nephrol.

[28] Ledergerber B, Furrer H, Rickenbach M, Lehmann R, Elzi L, Hirschel B, Cavassini M, Bernasconi E, Schmid P, Egger M, Weber R (2007). Swiss HIV Cohort Study: Factors associated with the incidence of type 2 diabetes mellitus in HIV-infected participants in the Swiss HIV Cohort Study. Clin Infect Dis.

[29] Bloomfield G, Hogan J, Keter A, Sang E, Carter J, Velasquez E, Kimaiyo S (2011). Hypertension and obesity among as cardiovascular risk factors among HIV seropositive patients in western Kenya. PLoS ONE.

[30] Somi G, Matee M, Makene CL, van den Hombergh J, Kilama B, Yahya-Malima KI, Masako P, Sando D, Ndayongeje J, Rabiel B, Swai RO (2009). Three years of HIV/AIDS care and treatment services in Tanzania: achievements and challenges. Tanzan J Health Res.

[31] Kothari CR (2004). Research Methodology, Methods and Techniques (Second Revised Edition).

[32] WHO (2007). Prevention of Cardiovascular Disease Pocket Guidelines for Assessment and Management of Cardiovascular Risk.

[33] WHO (2002). Non-Communicable Diseases in the South Asia Region.

[34] WHO (1995). Physical Status: The Use and Interpretation of Anthropometry. Report of a WHO Expert Committee WHO Technical Report Series 854.

[35] WHO (2008a). WHO STEPwise Approach to Surveillance (STEPS).

[36] WHO (2008b). Waist Circumference and Waist–Hip Ratio: Report of a WHO Expert Consultation Geneva.

[37] IDF (2006). The IDF Consensus Worldwide definition of the Metabolic Syndrome.

[38] WHO (1999). Definition, Diagnosis and Classification of Diabetes Mellitus and its Complications: Report of a WHO Consultation.

[39] Omech B, Sempa J, Castelnuovo B, Opio K, Otim M, Mayanja-Kizza H, Colebunders R, Manabe YC: *Prevalence of HIV-Associated Metabolic Abnormalities among Patients Taking First-Line Antiretroviral Therapy in Uganda*. ISRN AIDS 01/2012; 2012:960178. doi:10.5402/2012/960178 10.5402/2012/960178 PMC3767451 24052885

[40] Hurley E, Coutsoudis A, Giddy J, Knight SE, Loots E, Esterhuizen TM (2011). Weight evolution and perceptions of adults living with HIV following initiation of antiretroviral therapy in a South African urban setting. S Afr Med J.

[41] Zolopa A, Andersen J, Powderly W, Sanchez A, Sanne I, Suckow C, Hogg E, Komarow L (2009). Early antiretroviral therapy reduces AIDS progression/death in individuals with acute opportunistic infections: a multicenter randomized strategy trial. PLoS One.

[42] Mwangemi F, Lamptey P (2010). Integration of HIV and CVD Services in Kenya [Oral Presentation].

[43] UNAIDS report (2011). Chronic Care of HIV and Non Communicable Diseases; How to Leverage the HIV Experience?.

[44] Hejazi N, Huang MS, Lin KG, Choong LC (2013). Hypertension among HIV-infected adults receiving highly active antiretroviral therapy (HAART) in Malaysia. Glob J Health Sci.

[45] Puttawong S, Prasithsirikul W, Vadcharavivad S (2004). Prevalence of lipodystrophy in Thai-HIV infected patients. J Med Assoc Thail.

[46] Zannou DM, Denoeud L, Lacombe K, Amoussou Guenou D, Bashi J, Akakpo J, Girard PM (2009). Incidence of lipodystrophy and metabolic disorders in patients starting nonnucleoside reverse transcriptase inhibitors in Benin. International Medical Press.

[47] *At Work Issue: A Quarterly Publication of the Institute for Work & Health Issue 55 Winter*. 2009. http://www.iwh.on.ca/system/files/at-work/at_work_55.pdf: downloaded on 11^th^ September 2013

[48] Skovdal M, Campbell C, Madanhire C, Mupambireyi Z, Nyamukapa C, Gregson S: **Masculinity as a barrier to men's use of HIV services in Zimbabwe.** *Global Health* 2011.,**7**(13)**:** 10.1186/1744-8603-7-13 PMC3107786 21575149

[49] Herstad B (2010). Addressing Gender Issues Related to HIV Treatment Adherence Programs.

[CR50] The pre-publication history for this paper can be accessed here:http://www.biomedcentral.com/1471-2458/14/904/prepub

